# Exploring the complexity, treatment challenges, and outcomes in pediatric nodular lymphocyte predominant Hodgkin lymphoma: a perspective from a low–middle-income country

**DOI:** 10.3389/fonc.2024.1432650

**Published:** 2024-10-25

**Authors:** Youssef Madney, Amr Abdalla, Soha Ahmed, Marwa Romeih, Sally Fikry, Engy Mohammed, Hala Taha, Mohamed Zaghloul, Eman Attia

**Affiliations:** ^1^ Pediatric Oncology Department, National Cancer Institute, Cairo University, Cairo, Egypt; ^2^ Pediatric Oncology Department, Children’s Cancer Hospital Egypt, Cairo, Egypt; ^3^ Child Health Department, Sultan Qaboos University, Muscat, Oman; ^4^ Department of Clinical Oncology, Faculty of Medicine, Suez University, Suez, Egypt; ^5^ Radiodiagnosis Department, Faculty of Medicine, Helwan University, Cairo, Egypt; ^6^ Children’s Cancer Hospital Egypt, Cairo, Egypt; ^7^ Clinical Pharmacy Department, Children’s Cancer Hospital Egypt, Cairo, Egypt; ^8^ Clinical Research Department, Children’s Cancer Hospital Egypt, Cairo, Egypt; ^9^ Surgical Pathology Department, National Cancer Institute, Cairo University, Cairo, Egypt; ^10^ Radiation Oncology Department, National Cancer Institute, Cairo University, Cairo, Egypt

**Keywords:** children, nodular lymphocyte-predominant Hodgkin lymphoma, R-CHOP, ABVD, survival, relapse

## Abstract

**Background:**

In children, nodular lymphocyte-predominant Hodgkin lymphoma (NLPHL) is an uncommon subtype of Hodgkin lymphoma. Given the lack of data on the best chemotherapy regimen, there is a knowledge gap in best management.

**Methods:**

This retrospective study included all pediatric patients with NLPHL diagnosed and treated at the Children’s Cancer Hospital, Egypt (2007–2018). We analyzed the clinical characteristics, and treatment outcome according to disease stage (early and advanced), treatment strategy (doxorubicin, bleomycin, vinblastine, and dacarbazine [ABVD] or rituximab, cyclophosphamide, doxorubicin, vincristine, and prednisone [R-CHOP] and explored the prognostic factors for progression-free survival.

**Results:**

The median age at diagnosis was 12 years; 40 (68%) patients had early-stage disease, and 19 (32%) had advanced-stage disease. The median follow-up was 70 months; the 5-year EFS and OS were 75% and 97%, respectively. The 5-year EFS was 78% for early-stage disease and 60% for advanced-stage disease; the 5-year OS was 100% for early-stage disease and 88% for advanced-stage disease. The patients who underwent R-CHOP had a better 3-year EFS (100%) compared with those who underwent ABVD (65%) regimen (P = 0.01). Seventeen (25%) patients relapsed: 9/40 (22%) had early-stage disease, and 8/19 (42%) had advanced-stage disease. Splenic involvement, mediastinal disease, extranodal disease, and slow early response were independent predictors of relapse risk.

**Conclusion:**

Pediatric patients with early-stage NLPHL have an excellent prognosis, with a 5-year OS of 100%. However, those with advanced stages had a high relapse rate. R-CHOP was associated with a better response and relapse-free rate than ABVD.

## 1 Introduction

Nodular lymphocyte-predominant Hodgkin lymphoma (NLPHL) is an uncommon subtype of Hodgkin lymphoma (HL) occurring in children, accounting for 5%–10% of all pediatric cases of HL (1–3). NLPHL represents a distinct histopathological form of B-cell lymphoma, with a favorable prognosis (4, 5), and has clinical features such as localized disease at presentation, peripheral nodal involvement, and fewer unfavorable features (6, 7). In adults, the reported incidence of transformation to B-NHL ranges from 2% - to 17%. In contrast, the risk of transformation in children is unknown with splenic involvement, prior exposure to chemotherapy, and variant-pattern being the main risk factors (6, 8).

Pediatric patients with NLPHL with favorable features of early-stage disease have excellent outcomes with surgical resection only or with minimal chemotherapy. However, for patients with NLPHL with unfavorable features, such as advanced-stage disease, bulky disease, and B symptoms, their management is less clear due to their small numbers in clinical trials (9). Treatment with doxorubicin, bleomycin, vinblastine, and dacarbazine (ABVD) is associated with a high 10-year rate of recurrence and a histologic transformation into DLBCL of 40% (10). Given that clinical similarity to indolent B-NHL with an overall response rate of 100% after anti-CD20 antibody treatment with rituximab, treatment with rituximab, cyclophosphamide, doxorubicin, vincristine, and prednisone [R-CHOP] has been considered a promising alternative to the ABVD regimen (11, 12). Despite the lack of guidelines for the optimal therapeutic strategy for children with NLPHL, the outcomes tend to be excellent, with overall survival (OS) approaching 100% in most studies and event-free survival (EFS) varying depending on the therapy undergone (13–15).

There have been scant published data from Africa on NLPHL, including data on the best chemotherapy regimen and the impact of rituximab-containing regimens on the outcomes of children with NLPHL. We reported the clinical characteristics, prognostic factors for relapse, and treatment outcomes among 59 children with NLPHL diagnosed and treated at the Children’s Cancer Hospital Egypt (CCHE).

## 2 Patients and methods

This retrospective study included all newly diagnosed pediatric (≤ 18 years) patients with NLPHL treated in a large pediatric oncology center [Children’s Cancer Hospital, Egypt (CCHE)] in a low-middle-income country between (2007 -2018). Patients with NLPHL with ataxia telangiectasia or immunodeficiency were excluded. Of 1428 pediatric patients diagnosed with classic HL, 59 (4%) children were NLPHL. The disease staging was determined according to the Ann Arbor system (16), which included clinical history, physical examination, and positron emission tomography-computed tomography (PET-CT). The early stages were classified as stages I and II, and the advanced stages III and IV. A pathology review was done by two expert specialists in hemato-pathologists in our center for such rare disease entity and to rule out alternative diagnosis. Diagnosis based on both morphology and immunohistochemistry (ICH): Morphology based on characteristics of nodal tissue showed vague nodules of small lymphocytes with sparse and scattered large LP cells having multilobulated nuclei, finely granular chromatin, and variable small nucleoli. Immunohistochemical assessment based on the strong positive reaction of these large cells to CD20 and BCL6, while negative reaction to CD30 and CD15. CD20 and CD3 also stain reactive B and T lymphocytes in the background respectively with detected T cell rosettes surrounding large LP cells. CD21 is used to assure the presence of residual follicular dendritic cells within the neoplastic nodules, to exclude the possibility of T-cell/histiocyte-rich B-cell Non-Hodgkin lymphoma. This study was approved by the Scientific Advisory Committee (SMAC) and the Institutional Review Board (IRB) of our institution (Children Cancer Hospital Egypt). Informed consent was obtained before diagnostic assessment or therapy was initiated.

### 2.1 Definitions

Bulky disease was defined as a mediastinal nodal mass with a horizontal diameter of >1/3 the thoracic diameter or a single lymph node or continuous aggregate of nodal tissue outside the mediastinum measuring *>* 6* cm* in the longest diameter. The extranodal disease was defined as a localized disease arising in or extending to extra lymphatic sites (14). Relapse was documented by biopsy and radiological examination in all patients except one, whose relapse was based only on clinical and radiological progression.

### 2.2 Treatment strategy

**Figure 1:**
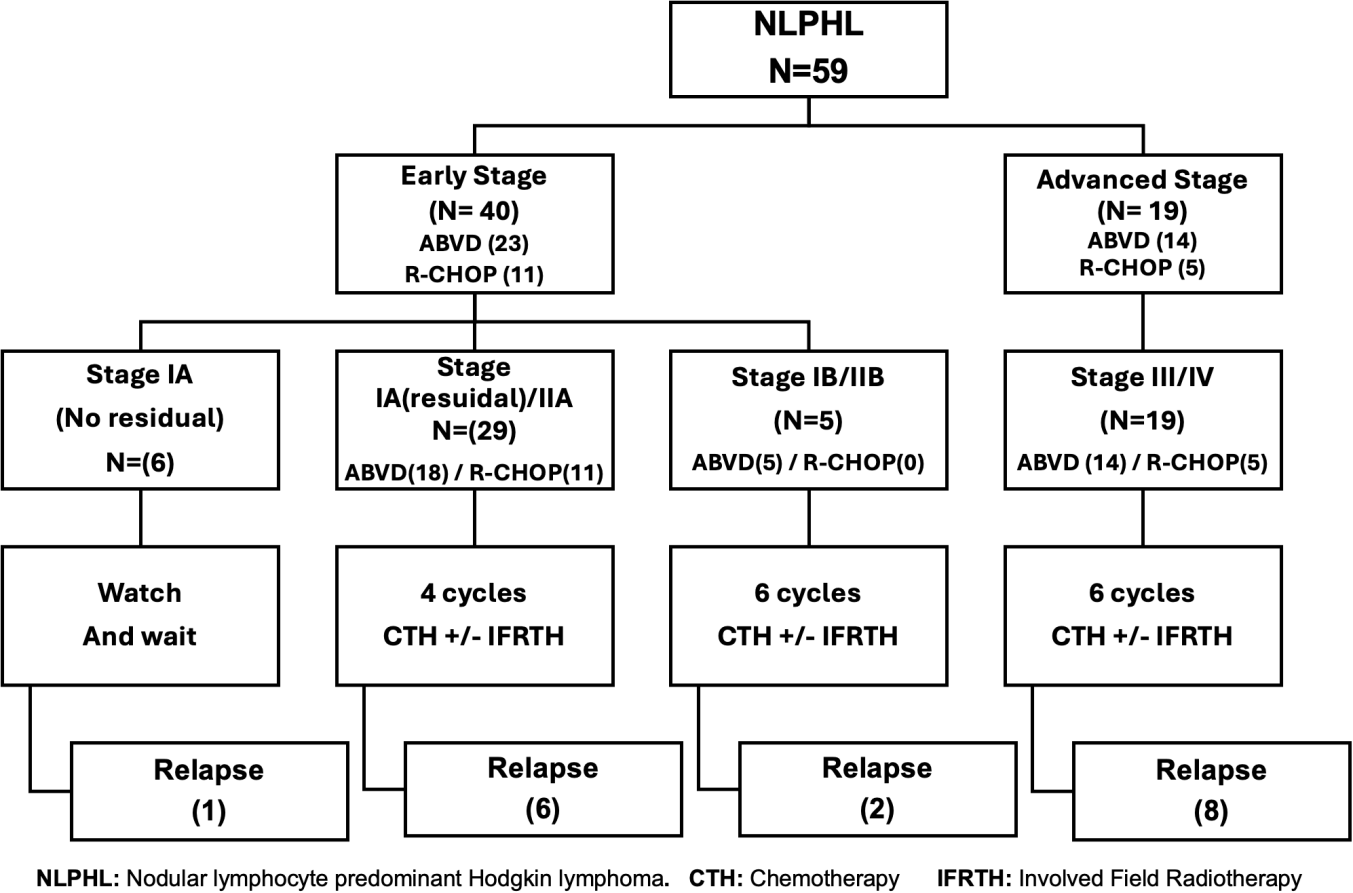
Treatment strategies for nodular lymphocyte-predominant Hodgkin lymphoma according to disease stage.

As a rare disease with limited data, we don’t have a standard protocol for NLPHL in children. Before 2015, patients with NLPHL were treated like classic HL with a combined modality treatment regimen (ABVD+/- IFRTH), after that we shifted to the R-CHOP regimen. Patients were treated according to the initial staging; those with surgically resected localized stage IA NLPHL without residual disease were closely observed. Patients with early-stage favorable disease (stage I or II without B symptoms, large mediastinal disease, or bulky disease) underwent 4 chemotherapy cycles. Patients with early unfavorable stage disease (B symptoms or bulky disease or large mediastinal disease) or advanced disease (stage III–IV) underwent 6 chemotherapy cycles. We assessed the patients after 2 cycles (early response assessment) and at the end of the chemotherapy (late response assessment). The choice of the investigator to add Involved field radiotherapy (IFRTH) has been made in > 50% of the patients, either low-risk or high-risk. In the era before Rituximab (2007-2014), we were treating patients with NLPHL as classic HL with a combined treatment approach (70%, (25/37) of patients received IFRTH (most of them with early-stage disease). After that, the indication for IFRTH was the inadequate response by PET-CT imaging after the end of chemotherapy. The dose of IFRTH was 1980 cGy (11 fractions), we applied to omit IFRTH for patients with CR after chemotherapy (50%, (8/16) received RTH, all those patients with advanced stage).” (**Figure 1**). Salvage chemotherapy for relapsed patients included ICE (Ifosfamide, carboplatin, and etoposide) regimen, with a total number of cycles of 4-6 cycles. If the patient achieved CR, then consolidation by auto-HSCT if indicated, Vinorelbine-Gemcitabine was used if additional chemotherapy needed to achieve a complete remission.

The indications for HSCT among our population group were; advanced stage at relapse, refractory disease, those with early relapse < 12 months from diagnosis, and late relapse if poor response to salvage treatment, or first-line treatment included a combined chemo-radiotherapy treatment approach.

### 2.3 Response evaluation

Definition of response: Complete response/remission (CR) was defined as the resolution of all clinical and radiological diseases with no residual extra mediastinal nodal mass of >2.0 cm and a negative fluorodeoxyglucose (Deauville score is less than 4). Partial response/remission (PR) was defined as a decrease of >50% in the sum of the product of the perpendicular diameters of the target nodes or extranodal lesions. Progressive disease was defined as a ≥50% increase in the product of the perpendicular diameter of any nodal mass lesion or new lesion progression (16, 17).

### 2.4 Statistical analysis

Descriptive statistics, frequency distributions, and percentages were calculated for the study participants’ baseline characteristics, outcome variables, and other covariates of interest as stage at diagnosis, response to treatment, and chemotherapy treatment regimen. Unpaired comparisons of categorical outcomes were performed with the Chi-square test. A two-sided probability of P < 0.05 was considered statistically significant. Survival curves for event-free survival (EFS) and overall survival (OS)were generated by the Kaplan–Meier method, and the log-rank statistic assessed the differences. Its measurement is calculated from the date of diagnosis to the date of the initial event for patients. The event includes disease relapse/progression or death. OS was defined as the time from diagnosis to death from any cause. The effects of different risk factors on relapse were tested using univariate Cox regression, and the statistically significant variables were reevaluated using a multivariate Cox regression model. Statistical analysis was performed using Statistical Package for SPSS, version 20.

## 3 Results

### 3.1 Patient characteristics

The clinical characteristics of the 59 children with confirmed NLPHL are shown in (**Table 1**). The median age of the entire cohort was 12 years (range, 5–18 years), with a male predominance (male: female,4:1). Forty patients (68%) had early-stage disease while 19 (32%) presented with advanced-stage disease. As regards unfavorable features; most of the patients with mediastinal disease involvement were advanced stage at diagnosis, B symptoms were found in 20% while only 2 (3%) patients had a bulky disease. Splenic involvement and extranodal disease at diagnosis were reported in (17%) and (13%) of the patients respectively.

**Table 1:** Clinical characteristics and treatment outcomes of 59 children with nodular lymphocyte-predominant Hodgkin lymphoma.

Total number of patients	59 (100%)
Age (5-18 y), median = 12	
Sex	
- Male - Female	48 (82%) 11 (18%)
Stage at diagnosis	
- Stage I - Stage II - Stage III - Stage IV	21 (35.5%) 19 (32%) 11 (19%) 8 (13.5%)
Early-stage disease	40 (68%)
- Stage IA - Stage IB - Stage II - Stage IIB	19 (32%) 2 (3%) 16 (27%) 3 (5%)
Advanced-stage disease	19 (32%)
- Stage III A - Stage III B/Bulky - Stage IV A - Stage IV B/Bulky	9 (15%) 2 (3%) 4 (6%) 4 (6%)
Clinical presentations	
- Mediastinal involvement - Stage II - Stage III - Stage IV - B symptoms - Splenic involvement - Extra-nodal disease - Bulky disease	13 (22%) 2 (3%) 3 (5%) 8 (13%) 11 (20%) 9 (17%) 8 (13%) 2 (3%)
Treatment	
**Surgery** **Chemotherapy** - ABVD - R-CHOP **Combined chemotherapy and RTH** - ABVD + RTH - R-CHOP + RTH	**6 (10%)** **20 (34%)** 12 (20%) 8 (13%) **33 (56%)** 25 (42%) 8 (13%)
**Relapse**	**17 (25%)**
Survival	
- **Alive** - **Died**	57 (97%) 2 (3%)

A, no B symptoms; RTH, Radiotherapy.

ABVD, Adriamycin, bleomycin, vinblastine, and dacarbazine.

R-CHOP, rituximab, cyclophosphamide, doxorubicin, vincristine, and prednisone.

### 3.2 Clinical features and treatment modalities among patients with early-stage disease

Out of the 40 patients, 5 had B symptoms and Mediastinal involvement was observed in 2 patients. Surgical excision was done in 6 (15%) patients and were kept under follow-up with only one patient relapsed. Among the remaining 34 patients, 23 received ABVD (17/23, were consolidated with IFRT (1980 cGy)) and 11 patients were treated with R-CHOP (3/11, received IFRT).

### 3.3 Clinical features and treatment modalities among patients with advanced-stage disease:

Most patients with advanced stage were associated with unfavorable clinical features: the most common was Mediastinal disease (n=11) followed by splenic involvement (n=9). Extranodal site involvement was reported in 11 patients (bone n=6, liver n=2, lungs n=2, bone marrow n=1). ABVD was given in 14 patients (8/14, consolidated by IFRT) and 5 underwent the R-CHOP regimen and received IFRT.

### 3.4 Toxicity

Chemotherapy was administered on an outpatient basis to all patients. From the ABVD arm, 4 patients had febrile neutropenia requiring admission and antibiotics. Cardiotoxicity with a decrease in cardiac contractility requiring treatment with angiotensin-converting enzyme inhibitors was reported in one patient who underwent ABVD and IFRT.

### 3.5 Relapse

The clinical features and outcomes of the relapsed patients were shown in (**Table 2**). Among the entire cohort, 17 patients experienced relapse (time to relapse ranging from 4–132 months) with no transformation or secondary DLBCL observed. Univariate Cox regression analysis showed that those with the mediastinal, splenic, and extranodal disease had significantly higher incidences of relapse (p=0.003, p<0.001 respectively). Regarding the initial stage at diagnosis, Stage IV was correlated with high relapse risk (p <0.001). Of great interest, early response assessment (after 2 cycles) was found to be a significant predictor of relapse (poor responders 8/12 vs good responders 9/47 with p-value <0.001). Using the treatment regimen, R-CHOP was associated with lower relapse risk as compared to ABVD regimen (0/16 for R-CHOP vs 17/37 for ABVD, p=0.002) (**Table 3**). In the early-stage disease group (n=40), 11 patients received R-CHOP regimen, with 3 of them consolidated with IFRTH (1980 cGy). No relapse was reported in this subgroup. The remaining 23 patients were treated with ABVD (17/23 and were consolidated with IFRT (1980 cGy). Among those, 8 patients experienced a relapse. For the advanced-stage disease group (n=19), 5 patients were treated with R-CHOP and received IFRT without any reported relapse after a median follow-up for 36 months. in contrast, 14 patients received ABVD, with 8 of them consolidated with IFRT. After a median follow-up of 70 months, 8 patients had a relapse in this subgroup (**Supplementary Table 1**). However, in multivariate analysis, no variables were statistically significant which may be explained by a small number of relapsed patients (**Supplementary Table 2**). All the relapsed patients received salvage chemotherapy, with 11 patients underwent autologous stem cell transplantation with no subsequent relapse reported.

**Table 2:** Treatment data of children with relapse NLPHL (N= 17).

Patient. Number	Stage	1st line Treatment	Response post 2nd cycle	Time to Relapse	Relapse Stage	Salvage 2nd line treatment	Response post 2nd cycle	Salvage 3rd line Treatment	Response post 2nd cycle	Auto-HSCT	Survival
6	IA	ABVD	PR	4M	II	ICE	CR			Yes	Alive
7	IIB	ABVD + RTH	CR	24M	III	ICE	PD	Vinorelbine- Gemcitabine	CR	Yes	Alive
8	IIB	ABVD + RTH	CR	28 M	II	ICE	CR			Yes	Alive
10	IA	ABVD	CR	33 M	I	ICE	CR			No	Alive
13	IA	Surgery	CR	34 M	I	R-CHOP	CR			No	Alive
18	IA	ABVD	CR	75 M	IV	ICE	PD	Vinorelbine - Gemcitabine	CR	Yes	Alive
20	IIA	ABVD + RTH	CR	46 M	III	ICE	CR			Yes	Alive
22	IA	ABVD + RTH	CR	12 M	II	ICE	CR			Yes	Alive
26	IIA	ABVD + RTH	CR	132M	III	ICE	CR			No	Alive
27	IVA	ABVD	PR	4 M	IV	ICE	CR			Yes	Alive
28	IVA	ABVD	PR	14 M	IV	ICE	PD	Vinorelbine - Gemcitabine	PD*	Yes	Alive
30	IVA	ABVD+RTH	PR	32 M	IV	ICE	CR			Yes	Alive
35	IVB	ABVD	PR	6 M	IV	ICE	PD	Vinorelbine - Gemcitabine	PD	–	Died**
40	IVB	ABVD	PR	6 M	IV	ICE	CR			–	Died***
42	IVB	ABVD	PR	6 M	IV	ICE	CR			Yes	Alive
43	IVB	ABVD	PR	24 M	IV	ICE	CR			Yes	Alive
47	IIIA	ABVD+RTH	CR	102 M	III	ICE	CR			No	Alive

PR, Partial response; PD, Progressive disease; CR, Complete remission; BMT, Bone Marrow Transplant.

*Patient had progressive disease post 3rd line then received 4th line R-DHAP (4 cycles with PET negative) so had auto-HSCT.

**Patient died from progressive disease after 2nd line (6 cycles ICE) and 3rd line (Vinorelbine -Gemcitabine).

***Patient died during supportive care of salvage ICE with gram-negative septicemia.

**Table 3:** Prognostic factors for relapse among children with nodular lymphocyte-predominant Hodgkin lymphoma.

		Total	Relapse		P value
			No	Yes	
**Clinical characteristics**		**59**	**42**	**17**	
Splenic involvement	Absent	50	40	10	**0.001**
	Present	9	2	7	
B symptoms	Absent	47	36	11	0.069
	Present	12	6	6	
Mediastinal involvement	Absent	46	37	9	**0.003**
	Present	13	5	8	
Bulky disease	Absent	57	42	15	0.079
	Present	2	0	2	
Extranodal disease	Absent	51	41	10	**0.001**
	Present	8	1	7	
Staging group					
	Stage I	21	16	5	1
	Stage II	19	15	4	
	Stage III	11	10	1	**0.001**
	Stage IV	8	1	7	
Response after 2 cycles					
	CR	47	38	9	**0.001**
	PR	12	4	8	
Therapeutic strategy					
	**Chemotherapy**	**20**	**11**	**9**	0.06
	ABVD	12	3	9	
	R-CHOP	8	8	0	
	**CMT**	**33**	**26**	**7**	
	ABVD + RTH	25	18	7	
	R-CHOP + RTH	8	8	0	
	**Surgery**	**6**	**5**	**1**	
Therapeutic regimen					
	**R-CHOP**	16	16	0	**0.002**
	**ABVD**	37	21	16	

CR, complete response; PR, partial response; CMT, combined modality treatment.

RTH, radiotherapy; ABVD, adriamycin, bleomycin, vinblastine,dacarbazine.

R- CHOP, rituximab, prednisolone, doxorubicin, cyclophosphamide, vincristine.

### 3.6 Survival

**Figure 2:**
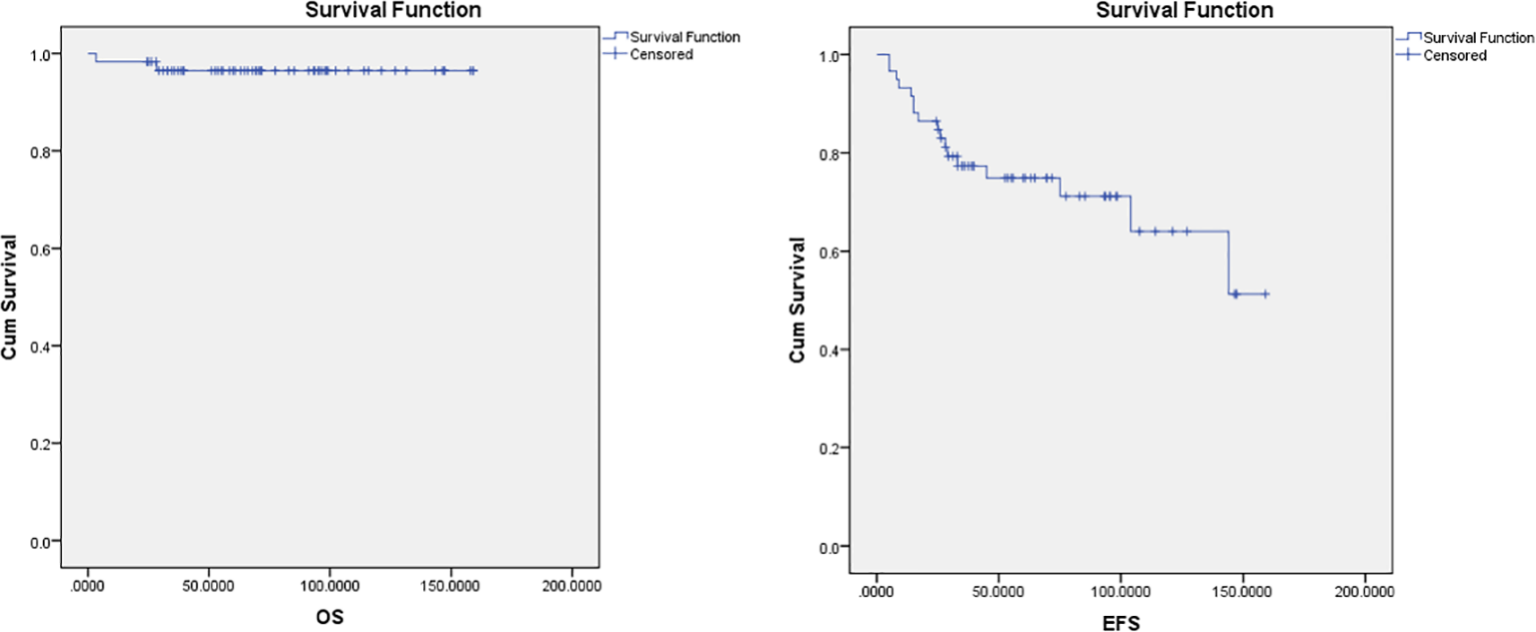
Overall survival (OS) and Event-free survival (EFS) for the pediatric NLPHL patients.

**Figure 3:**
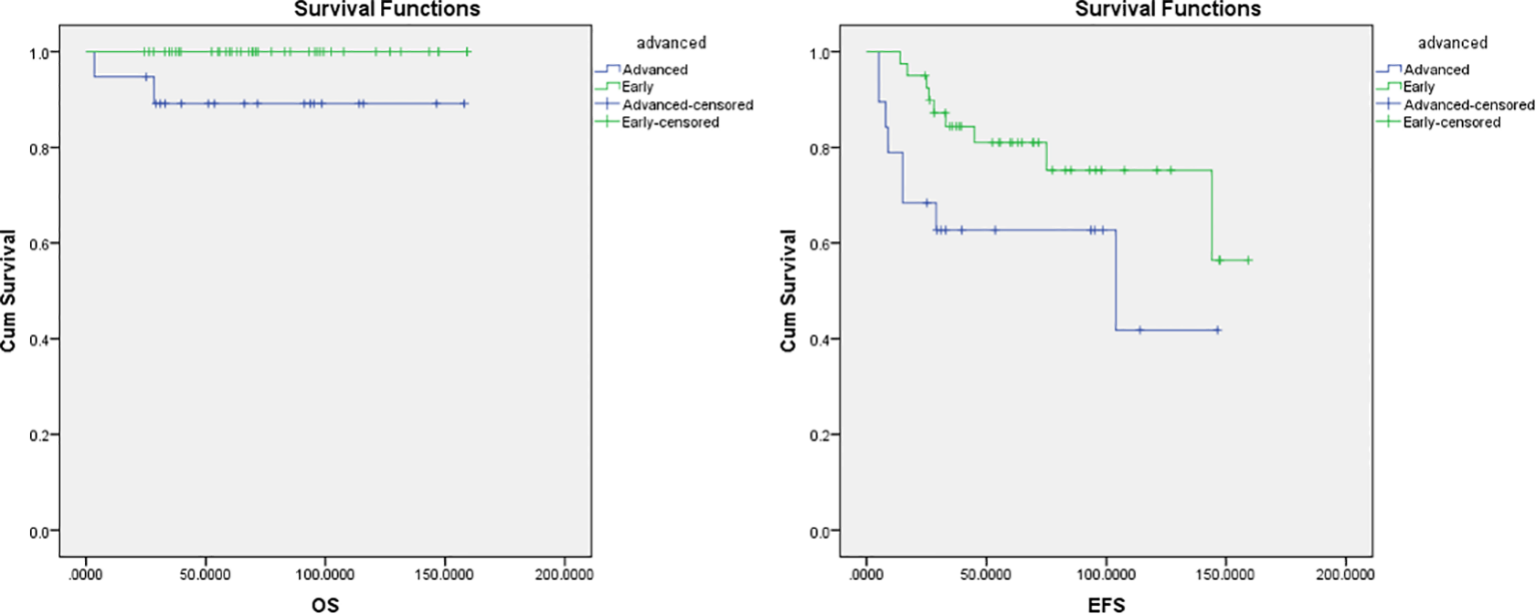
Overall survival (OS) and Event-free survival (EFS) for the early and advanced stage NLPHL.

**Figure 4:**
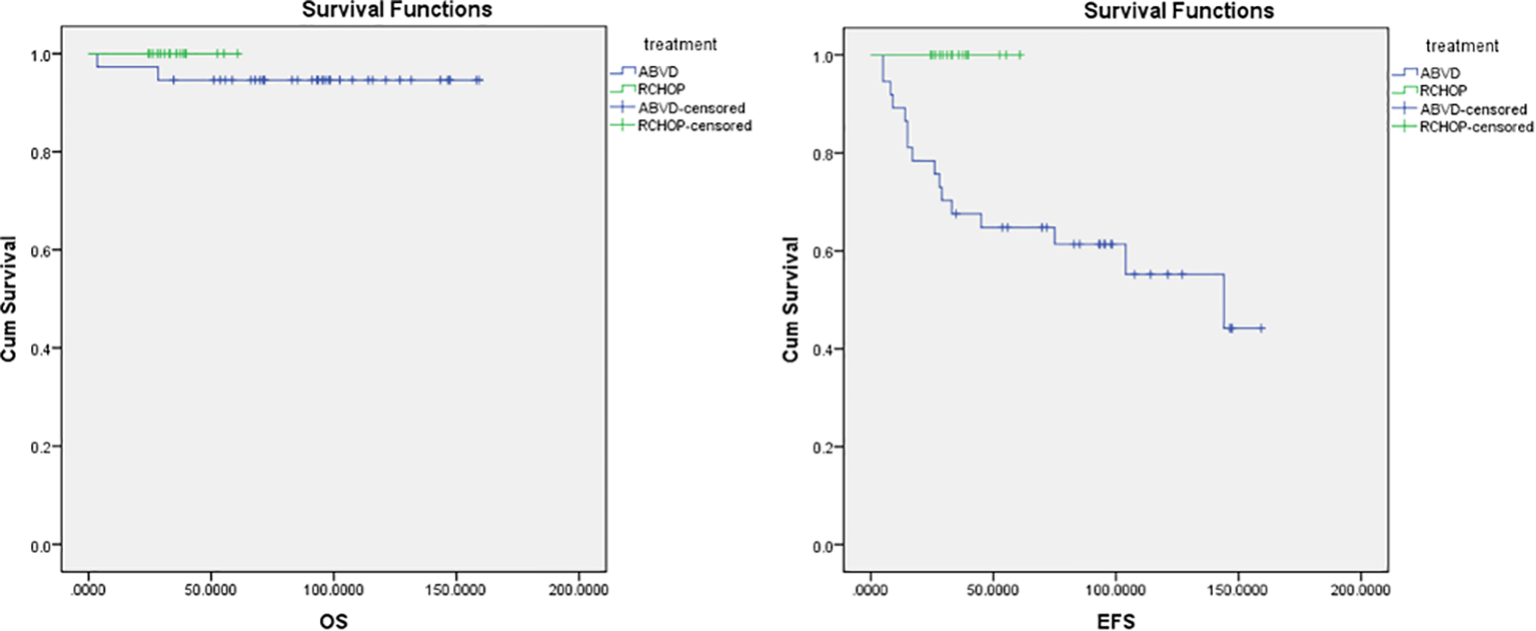
Overall survival (OS) and event-free survival (EFS) for ABVD and R-CHOP regimens. ABVD, Doxorubicin, Bleomycin, Vinblastine, and Dacarbazine; CMT, Combined Modality Treatment; COG, Children’s Oncology Group; CTH, Chemotherapy, CR, Complete Response; EFS, Event Free Survival; FDG-PET, Fluorodeoxyglucose–positron emission tomography; HL, Hodgkin lymphoma; IFRT, Involved-field radiotherapy; NLPHL, Nodular Lymphocyte-Predominant Hodgkin Lymphoma; NHL, Non-Hodgkin Lymphoma; OS, Overall Survival; PD, Progressive Disease; PPD, Product of perpendicular diameters; PR, Partial Response; R-CHOP, Rituximab, Cyclophosphamide, Doxorubicin, Vincristine, and Prednisone.

The median follow-up period was 70 months in ABVD group and 36 months for R-CHOP group. The 5-year EFS and OS for the whole study group were 75% and 97%, respectively (**Figure 2**). Patients with advanced-stage had significantly lower 5-year OS (88% vs 100%, p=0.03) and EFS (60% vs 78%, p=0.03) than those with early-stage disease (**Figure 3**). In our study analysis, patients who received combined modality therapy exhibited a lower relapse rate (21% [7/33] compared to 45% [9/20]) than the chemotherapy group), reflecting improved 5-year disease-free survival (P = 0.01). However, the combined modality did not significantly affect 5-year overall survival (P = 0.12) (**Supplementary Figure 1**). Patients who received R-CHOP had a better 3-year EFS compared with ABVD (100% versus 65%, P = 0.04) (**Figure 4**). Two relapsed patients died, one due to progressive disease and the second because of sepsis during salvage chemotherapy.

## 4 Discussion

Nodular Lymphocyte Predominant (NLPHL) is a rare subtype of Hodgkin lymphoma (HL). Despite its rarity, NLPHL presents distinct clinical features and treatment challenges compared to classical HL. Understanding these features and treatment outcomes is crucial for optimizing patient management. Given the lack of data on the best chemotherapy regimen, there is a knowledge gap in the standard care of management. Our study aimed to describe the clinical features, prognostic factors for relapse, and treatment outcome of pediatric patients with NLPHL treated at a large oncology center in a low-middle income country.

Previous studies reported that the survival of patients with early-stage NLPNL disease exceeds 90% (13, 14, 18). Given excellent long-term survival rates, more effort was focused on limiting treatment-related toxicities and improving quality of life. Some advocate for a watch-and-wait approach in asymptomatic patients with low tumor burden. The Children’s Oncology Group (COG) conducted a chemotherapy-sparing study with 75% of highly selected patients (stage 1A NLPHL) after surgical resection only; all relapsed patients (13 of 52) were salvaged effectively with chemotherapy, with an OS of 100% (14). Furthermore, data from the European network group on pediatric Hodgkin lymphoma (19) among 58 children with early-stage NLPHL who underwent surgical resection reported that a substantial proportion of patients experienced long-term remission with PFS was 67% for the CR group with OS 100% when complete resection was achieved. This aligns with the current results in which long-term remission was reported in 5/6 patients treated with surgery alone, making this chemotherapy-sparing strategy an attractive option for these patients. A short course of low-intensive chemotherapy such as R-CVP (4 cycles) may be an attractive treatment option for unresected early-stage disease. Similarly, these results showed that patients who received R-CHOP regimen had an excellent outcome.

Advanced-stage nodular lymphocyte-predominant Hodgkin lymphoma is very rare. Compared with early-stage disease, this is a biologically aggressive disease with a poor response and a higher relapse rate. There is no consensus regarding the optimal treatment for this disease, although outcomes for patients are excellent when they are treated according to standard regimens for intermediate-risk or high-risk Hodgkin lymphoma (15). However, Xing et al. reported that ABVD might be inadequate for initial therapy for patients with advanced NLPHL and is associated with a higher recurrence rate and histologic transformation into aggressive B-NHL (10). Also, Shankar et al. reported that the pediatric advanced-stage NLPHL relapse rate was double for chemotherapy only when compared with rituximab plus chemotherapy (15% [3/20] vs. 8% [1/12]), taking into consideration a small number of patients and the retrospective nature of the study (20). This is in line with the current results where patients with advanced stage who underwent a rituximab-containing regimen showed better outcomes (**Figure 4**) and lower relapse risk (0/5 relapsed in the R-CHOP vs. 8/14 in the ABVD group).

In our center, pediatric nLPHL was treated initially as classic HL. Radiotherapy was delivered in around 56% of the patients in combination with high cumulative doses of anthracyclines (ABVD), although the internationally accepted treatment paradigm in pediatric nLPHL treatment is to spare chemotherapy (and radiotherapy) as much as possible in this highly curable entity. Excellent survival rates of close to 100% are reported throughout all consortia and available trials. Similarly, in this study the combined modality had no significant impact on 5-year OS, emphasizing the strategy of radiation-sparing therapy.

Although early response assessment by FDG-PET has been established in classic HL, there is limited evidence for the use of PET-CT during treatment to inform treatment decisions in either adults or children with NLPHL (21, 22). COG protocol (AHOD03P1) omitted RTH for pediatric patients with NLPHL achieving a CR after 3 cycles of AVPC (92% avoided upfront RTH) with excellent overall survival (14). Interestingly, our results showed that early response assessment by PET-CT had a significant impact on the relapse rate (9/47 vs 8/12. P value <0.001). This goes with a recent recommendation from the Hodgkin lymphoma subgroup of the UK National Cancer Research Institute, which recommends a response-adapted approach using low-dose involved-site radiotherapy (ISRT) for patients who have an inadequate response to first-line chemotherapy is a better setting for CMT in patients with early-stage pediatric NLPHL (23).

Although NLPHL has a better overall disease outcome than cHL, several studies have identified worse prognosis with specific risk factors including advanced-stage disease, presence of B symptoms (10), mediastinal lymphadenopathy, splenic involvement, and extra-nodal sites such as liver and/or bone marrow involvement (24). This is consistent with the current results where a higher incidence of relapse was observed among patients with splenic, mediastinal, and extra-nodal disease involvement (p<0.001). Variant histology has been shown to be an independent predictor of poor treatment outcome, advanced-stage disease, and higher risk of recurrent disease in both children and adults. Despite the lack of data about histological variants among our cohort, a previously published study conducted in our institution reported that (25) variant histology was associated with unfavorable clinical characteristics, such as B-symptoms, advanced stage and extranodal disease with 39% of patients with variant histology experienced disease progression or relapse, compared to only 15% of patients with typical patterns. That the presence of unfavorable risk features warrants a more intensive treatment is still controversial and additional analyses are necessary to include it in risk classification and treatment intensification.

Given the rarity of relapsed or primary refractory nLPHL in children and adolescents, data on treatment outcomes are limited and the conduct of randomized trials is challenging. Several factors including relapse histology, prior therapy, type of chemotherapy regimen, and duration of first remission should be taken into consideration (20). A study conducted by Shankar et al., reported that 94% of children diagnosed with NLPHL (including both relapsed cases and poor responders) were effectively treated with second-line chemotherapy or radiotherapy (20). Our analysis revealed that 25% of patients experienced a relapse, with 90% of them responding positively to second-line treatment. Notably,11 patients achieved successful outcomes through autologous stem cell transplantation following salvage chemotherapy. These findings emphasize the importance of tailored treatment approaches for NLPHL patients.

The global nLPHL one working group (GLOW) highlighted in a recent survey that the rarity of this disease, less availability of expert hematopathologists and fewer PET resources may make adapted treatment protocols difficult to execute. A prospective trial is still needed for the development of adapted treatment regimens in LMICs (26). We had a reasonable number of patients and an adequate follow-up duration. The main limitations of our study were the nonuniform treatment of patients either to be treated like HL or by a different strategy, radiotherapy consolidation was allowed to be given at the investigator’s discretion and was not applied in a response-based fashion.

In conclusion, children with NLPHL had highly favorable outcomes. Those with early-stage disease had excellent survival, and more effort should be focused on limiting treatment-related toxicity. Surgical resection can spare chemotherapy in a select group of patients. A short course of low-intensive chemotherapy such as R-CVP (3-4 cycles) may be an attractive treatment option for early-stage disease (unresected stage IA or IIA NLPHL). The patients with advanced-stage disease still had good outcomes; however, the question remains about the optimal regimen. Our data support a potential role for R-CHOP chemotherapy in NLPHL, given that early data showed excellent response rates and remission among our patients. Furthermore, incorporating prognostic unfavorable risk features (B symptoms, mediastinal disease, splenic involvement, and variant histology) along with early response assessments in risk classification and treatment intensification should guide treatment decisions. Long-term follow-up, a larger number of patients, and international collaborative studies are still needed to figure out the optimal treatment for this rare disease.

## Data Availability

The datasets presented in this study can be found in online repositories. The names of the repository/repositories and accession number(s) can be found in the article/**Supplementary Material**.

## References

[1] Sandoval C Venkateswaran L Billups C Slim M Jayabose S Hudson MM. Lymphocyte-predominant hodgkin disease in children. J Pediatr Hematol Oncol. (2002) 24:269–73. doi: 10.1097/00043426-200205000-00010 11972094

[2] Murphy SB Morgan ER Katzenstein HM Kletzel M. Results of little or no treatment for lymphocyte-predominant Hodgkin disease in children and adolescents. J Pediatr Hematol Oncol. (2003) 25:684–7. doi: 10.1097/00043426-200309000-00003 12972802

[3] Pellegrino B Terrier-Lacombe MJ Oberlin O Leblanc T Perel Y Bertrand Y. Lymphocyte-predominant Hodgkin’s lymphoma in children: Therapeutic abstention after initial lymph node resection - A study of the French Society of Pediatric Oncology. J Clin Oncol. (2003) 21:2948–52. doi: 10.1200/JCO.2003.01.079 12885814

[4] Hartmann S Eichenauer DA. Nodular lymphocyte predominant Hodgkin lymphoma: pathology, clinical course and relation to T-cell/histiocyte rich large B-cell lymphoma. Pathology. (2020) 52:142–53. doi: 10.1016/j.pathol.2019.10.003 31785822

[5] Spinner MA Varma G Advani RH. Modern principles in the management of nodular lymphocyte-predominant Hodgkin lymphoma. Br J Haematol. (2019) 184:17–29. doi: 10.1111/bjh.15616 30485408

[6] Shankar A Daw S. Nodular lymphocyte predominant Hodgkin lymphoma in children and adolescents - a comprehensive review of biology, clinical course and treatment options. Br J Haematol. (2012) 159:288–98. doi: 10.1111/bjh.12055 22994199

[7] Prasad M Narula G Chinnaswamy G Arora B Shet T Panjwani P. Unfavorable presentation but comparable outcome: Presentation and outcome of children with nodular lymphocyte predominant Hodgkin lymphoma from India. Pediatr Blood Cancer. (2018) 65:1–7. doi: 10.1002/pbc.27288 29893471

[8] Huang JZ Weisenburger DD Vose JM Greiner TC Aoun P Chan WC. Diffuse large B-cell lymphoma arising in nodular lymphocyte predominant hodgkin lymphoma: a report of 21 cases from the nebraska lymphoma study group. Leuk Lymphoma. (2004) 45:1551–7. doi: 10.1080/1042819031000149421 15370206

[9] Marks LJ Pei Q Bush R Buxton A Appel B Kelly KM. Outcomes in intermediate-risk pediatric lymphocyte-predominant Hodgkin lymphoma: A report from the Children’s Oncology Group. Pediatr Blood Cancer. (2018) 65:1–8. doi: 10.1002/pbc.27375 PMC6192844 30277639

[10] Xing KH Connors JM Lai A Al-Mansour M Sehn LH Villa D. Advanced-stage nodular lymphocyte predominant Hodgkin lymphoma compared with classical Hodgkin lymphoma: a matched pair outcome analysis. Blood. (2014) 123:3567–73. doi: 10.1182/blood-2013-12-541078 24713929

[11] Eichenauer DA Fuchs M Pluetschow A Klimm B Halbsguth T Böll B. Phase 2 study of rituximab in newly diagnosed stage IA nodular lymphocyte-predominant Hodgkin lymphoma: a report from the German Hodgkin Study Group. Blood. (2011) 118:4363–5. doi: 10.1182/blood-2011-06-361055 21828141

[12] Advani RH Horning SJ Hoppe RT Daadi S Allen J Natkunam Y. Mature results of a phase II study of rituximab therapy for nodular lymphocyte–predominant hodgkin lymphoma. J Clin Oncol. (2014) 32:912–8. doi: 10.1200/JCO.2013.53.2069 24516013

[13] Hall GW Katzilakis N Pinkerton CR Nicolin G Ashley S McCarthy K. Outcome of children with nodular lymphocyte predominant Hodgkin lymphoma - a Children’s Cancer and Leukaemia Group report. Br J Haematol. (2007) 138:761–8. doi: 10.1111/j.1365-2141.2007.06736.x 17760808

[14] Appel BE Chen L Buxton AB Hutchison RE Hodgson DC Ehrlich PF. Minimal treatment of low-risk, pediatric lymphocyte-predominant hodgkin lymphoma: A report from the children’s oncology group. J Clin Oncol. (2016) 34:2372–9. doi: 10.1200/JCO.2015.65.3469 PMC4981978 27185849

[15] Shankar AG Roques G Kirkwood AA Lambilliotte A Freund K Leblanc T. Advanced stage nodular lymphocyte predominant Hodgkin lymphoma in children and adolescents: clinical characteristics and treatment outcome - a report from the SFCE & CCLG groups. Br J Haematol. (2017) 177:106–15. doi: 10.1111/bjh.14518 28220934

[16] Lister TA Crowther D Sutcliffe SB Glatstein E Canellos GP Young RC. Report of a committee convened to discuss the evaluation and staging of patients with Hodgkin’s disease: Cotswolds meeting. J Clin Oncol. (1989) 7:1630–6. doi: 10.1200/JCO.1989.7.11.1630 2809679

[17] Cheson BD Fisher RI Barrington SF Cavalli F Schwartz LH Zucca E. Recommendations for initial evaluation, staging, and response assessment of hodgkin and non-hodgkin lymphoma: The lugano classification. J Clin Oncol. (2014) 32:3059–67. doi: 10.1200/JCO.2013.54.8800 PMC4979083 25113753

[18] Shankar AG Kirkwood AA Hall GW Hayward J O’Hare P Ramsay AD. Childhood and Adolescent nodular lymphocyte predominant Hodgkin lymphoma - A review of clinical outcome based on the histological variants. Br J Haematol. (2015) 171:254–62. doi: 10.1111/bjh.13540 26115355

[19] Mauz-Körholz C Gorde-Grosjean S Hasenclever D Shankar A Dörffel W Wallace WH. Resection alone in 58 children with limited stage, lymphocyte-predominant Hodgkin lymphoma–experience from the European network group on pediatric Hodgkin lymphoma. Cancer. (2007) 110:179–85. doi: 10.1002/cncr.22762 17526010

[20] Shankar AG Kirkwood AA Depani S Bianchi E Hayward J Ramsay AD. Relapsed or poorly responsive nodular lymphocyte predominant Hodgkin lymphoma in children and adolescents - a report from the United Kingdom’s Children’s Cancer and Leukaemia Study Group. Br J Haematol. (2016) 173:421–31. doi: 10.1111/bjh.13979 26996288

[21] Hutchings M Loft A Hansen M Ralfkiaer E Specht L. Different histopathological subtypes of Hodgkin lymphoma show significantly different levels of FDG uptake. Hematol Oncol. (2006) 24:146–50. doi: 10.1002/hon.782 16729353

[22] Fanale MA Cheah CY Rich A Medeiros LJ Lai CM Oki Y. Encouraging activity for R-CHOP in advanced stage nodular lymphocyte–predominant Hodgkin lymphoma. Blood. (2017) 130(4):472–7. doi: 10.1182/blood-2017-02-766121 PMC5578726 28522441

[23] Shankar A Hall GW McKay P Gallop-Evans E Fielding P Collins GP. Management of children and adults with all stages of nodular lymphocyte predominant Hodgkin lymphoma: A consensus-based position paper from the Hodgkin lymphoma subgroup of the National Cancer Research Ins. Br J Haematol. (2022) 197:679–90. doi: 10.1111/bjh.18169 35362554

[24] Eichenauer DA Engert A. How I treat nodular lymphocyte predominant Hodgkinlymphoma. Blood. (2020) 136(26):29872993. doi: 10.1182/blood.2019004044 32877522

[25] Ali N Moussa E Khorshed E Zaghloul MS Elnashar A Abdalla A. Variant histology of pediatric nodular lymphocyte-predominant Hodgkin lymphoma with IgD and CD30 expression. Pediatr Blood Cancer. (2023) 70(11):e30647. doi: 10.1002/pbc.30647 37638819

[26] Lo AC Major A Super L Appel B Shankar A Constine LS. Practice patterns for the management of nodular lymphocyte-predominant Hodgkin lymphoma (NLPHL): an international survey by the Global NLPHL One Working Group (GLOW). Leukemia Lymphoma. (2022) 63:1997 2000. doi: 10.1080/10428194.2022.2053533 35357263

