# Supplementary material for: Exploring the complexity, treatment challenges, and outcomes in pediatric nodular lymphocyte predominant Hodgkin lymphoma: a perspective from a low–middle-income country
Source: Front Oncol. 2024 Oct 25;14:1432650. doi: 10.3389/fonc.2024.1432650 (PMC11543585; doi:10.3389/fonc.2024.1432650)
Supplement: Supplementary file 1 [file DataSheet1.docx]

**Table (1) Supp: Treatment strategies, response, and relapse according to disease risk staging of 59 patients with nodular lymphocyte-predominant Hodgkin lymphoma**

| **NLPHL patients**  **(n = 59)** | **Surgery**  **(n = 6)** | **Chemotherapy**  **(n = 20)** | | **Combined modality therapy**  **(n = 33)** | |
| --- | --- | --- | --- | --- | --- |
| **Early-stage (n = 40)** | **Surgery**  **(n = 6)** | **ABVD**  **(n = 6)** | **R-CHOP**  **(n = 8)** | **ABVD + IFRTH**  **(n = 17)** | **R-CHOP + IFRTH**  **(n = 3)** |
| Early response assessment after 2 cycles |  | CR (n = 5)  PR (n = 1) | CR (n = 8) | CR (n = 15)  PR (n = 2) | CR (n = 3) |
| Relapse (n = 9) | 1 | 3 | 0 | 5 | 0 |
| **Advanced-stage (n = 19)** |  | **ABVD**  **(n = 6)** | **R-CHOP**  **(n = 0)** | **ABVD + IFRTH**  **(n = 8)** | **R-CHOP + IFRTH**  **(n = 5)** |
| Early response assessment after 2 cycles |  | CR (n = 1)  PR (n = 5) | - | CR (n = 7)  PR (n = 1) | CR (n = 4)  PR (n = 1) |
| Relapse (n = 8) |  | 6 | - | 2 | 0 |

**Early-stage**: (stage I and II); **Advanced-stage**: stage III and IV; **IFRTH**: involved field radiotherapy; **ABVD**: Adriamycin, bleomycin, vinblastine, dacarbazine;

**R-CHOP**: rituximab, prednisolone, doxorubicin, cyclophosphamide, vincristine; **CR**: complete remission; **PR**: partial response

**Table (2) Supp: Multivariate analysis for prognostic factors for relapse among children with NLPHL**

| Model | Sig. | 95.0% Confidence Interval for B | |
| --- | --- | --- | --- |
|  |  | Lower Bound | Upper Bound |
| **Bulky** | 0.343 | -.374 | 1.047 |
| **Age** | 0.606 | -.027 | .046 |
| **Sex** | 0.341 | -.517 | .184 |
| **B symptoms** | 0.144 | -.084 | .548 |
| **Mediastinal involvement** | 0.630 | -.380 | .619 |
| **Spleen** | 0.414 | -.432 | 1.026 |
| **Early versus advanced stage** | 0.509 | -.502 | .253 |
| **Radiotherapy** | 0.037 | -.615 | -.020 |
| **ABVD/RCHOP** | 0.180 | -.420 | .082 |

**
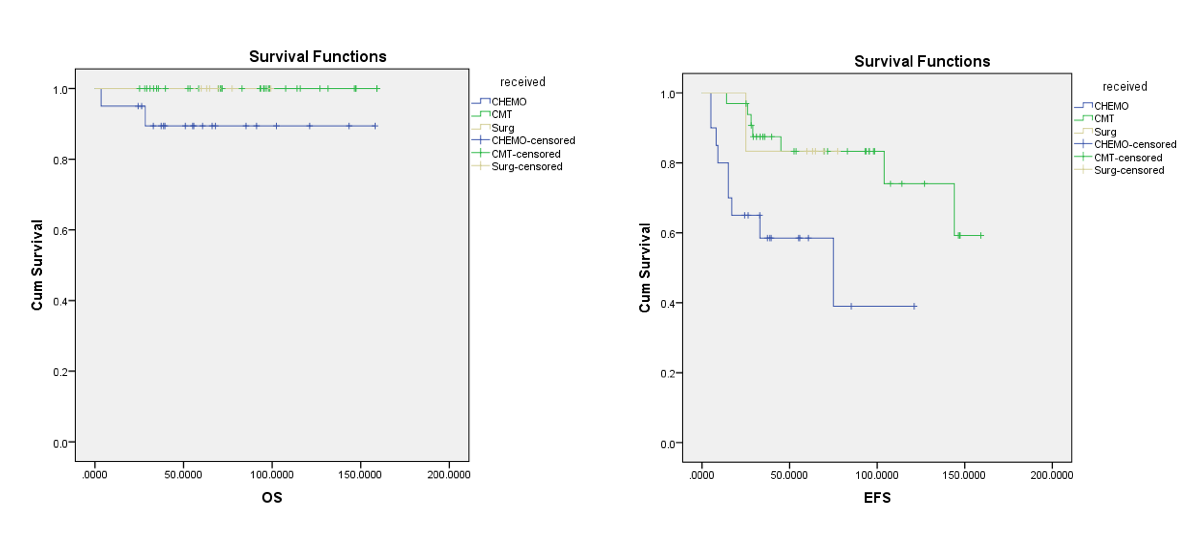
**

**Figure (1) Supp: Overall survival (OS) and event-free survival (EFS) for chemotherapy and combined modality treatment groups**
